# Online prediction model for primary aldosteronism in patients with hypertension in Chinese population: A two-center retrospective study

**DOI:** 10.3389/fendo.2022.882148

**Published:** 2022-08-02

**Authors:** Wenbin Lin, Wenjia Gan, Pinning Feng, Liangying Zhong, Zhenrong Yao, Peisong Chen, Wanbing He, Nan Yu

**Affiliations:** ^1^ Department of Laboratory Medicine, Zhujiang Hospital, Southern Medical University, Guangzhou, China; ^2^ Department of Clinical Laboratory, The First Affiliated Hospital of Sun Yat-Sen University, Guangzhou, China; ^3^ Department of Cardiology, Sun Yat-Sen Memorial Hospital of Sun Yat-Sen University, Guangzhou, China; ^4^ Department of Medical Laboratory, School of Laboratory Medicine and Biotechnology, Southern Medical University, Guangzhou, China

**Keywords:** primary aldosteronism, online prediction model, risk factors, hypertension, primary care

## Abstract

**Background:**

The prevalence of primary aldosteronism (PA) varies from 5% to 20% in patients with hypertension but is largely underdiagnosed. Expanding screening for PA to all patients with hypertension to improve diagnostic efficiency is needed. A novel and portable prediction tool that can expand screening for PA is highly desirable.

**Methods:**

Clinical characteristics and laboratory data of 1,314 patients with hypertension were collected for modeling and randomly divided into a training cohort (919 of 1,314, 70%) and an internal validation cohort (395 of 1,314, 30%). Additionally, an external dataset (n = 285) was used for model validation. Machine learning algorithms were applied to develop a discriminant model. Sensitivity, specificity, and accuracy were used to evaluate the performance of the model.

**Results:**

Seven independent risk factors for predicting PA were identified, including age, sex, hypokalemia, serum sodium, serum sodium-to-potassium ratio, anion gap, and alkaline urine. The prediction model showed sufficient predictive accuracy, with area under the curve (AUC) values of 0.839 (95% CI: 0.81–0.87), 0.814 (95% CI: 0.77–0.86), and 0.839 (95% CI: 0.79–0.89) in the training set, internal validation, and external validation set, respectively. The calibration curves exhibited good agreement between the predictive risk of the model and the actual risk. An online prediction model was developed to make the model more portable to use.

**Conclusion:**

The online prediction model we constructed using conventional clinical characteristics and laboratory tests is portable and reliable. This allowed it to be widely used not only in the hospital but also in community health service centers and may help to improve the diagnostic efficiency of PA.

## Introduction

Primary aldosteronism (PA) is the most common cause of secondary hypertension, characterized by high blood pressure, decreased plasma renin, and excessive aldosterone secretion from one or both the glomerulosa zona of the adrenal cortex (1–4). The prevalence of PA varies from 5% to 20% (5–7). Excess aldosterone leads to an increase in target organ damage, such as kidney, heart and vasculature (8–16), and cardiovascular and cerebrovascular events including atrial fibrillation, arrhythmia, ventricular hypertrophy, heart failure, stroke, and cerebral infarction (9). PA can be effectively treated by mineralocorticoid receptor antagonist or laparoscopic adrenalectomy (17, 18); thus, it is important for early identification using an easy and reliable method.

Measurement of plasma aldosterone concentrations and renin activity to assess the plasma aldosterone-to-renin ratio (ARR) is the most recommended screening method for PA (18–20). It required that medications, which interfere with the renin–angiotensin system, especially those that may stimulate renin secretion, should be withdrawn for at least 2–4 weeks prior to testing (18). Although the Endocrine Society guideline provided a recommendation to screen for patients with hypertension with an increased risk of PA (18, 21), it encompasses only 50%~60% of patients with hypertension. However, the fact is that less than 3% and 8% of patients were actually screened in the United States and Europe, respectively (22–25). Additionally, in China, screening PA through ARR is difficult to popularize due to the lack of professional equipment in healthcare centers, community hospitals, and even some municipal hospitals. Therefore, a novel and portable prediction tool that can expand screening for PA is highly desirable.

Medical applications of artificial intelligence and machine learning, particularly in disease prediction and prognostic prediction, have made remarkable progress (26–28). Previous studies have successfully applied machine learning for the prediction of PA in Italy (22) and subtype diagnosis and clinical outcomes after adrenalectomy in PA (29, 30). Herein, we aim to build and validate an online prediction model based on supervised learning algorithms using a new dataset in the Chinese population to predict the probability of PA in patients with hypertension.

## Method

### Study population

Consecutive patients definitely diagnosed with essential hypertension (EH) and PA from The First Affiliated Hospital, Sun Yat-sen University, were included in the study between January 2018 and December 2020. Furthermore, we also collected an external validation set from Sun Yat-sen Memorial Hospital, Sun Yat-sen University, between January 2020 and December 2020.

Patients with PA were diagnosed according to Endocrine Society and European Society of Hypertension recommendations (18, 20). A threshold value of ARR of 30, together with aldosterone concentration > 10 ng/dl, was considered as suspected positive PA. Further confirmatory tests such as intravenous saline-loading test or captopril-challenge test were performed in patients with positive screening. Patients with an ARR greater than 30 after the captopril challenge test or aldosterone greater than 5 ng/dl after an intravenous saline-loading test were diagnosed as PA. However, guidelines also suggested that there was no need for further confirmatory testing in patients with spontaneous hypokalemia, plasma aldosterone level > 20 ng/dl plus plasma renin level below detection levels. Drugs that could interfere with the ARR (including aldosterone receptor antagonists, potassium-sparing diuretics, and non-steroidal anti-inflammatory drugs) were withdrawn or changed for medications that have minimal impact on ARR (such as non-central α-receptor blockers and non-dihydropyridine calcium ion antagonists) before screening test. Hypokalemia was corrected before the assessment of ARR. Patients with EH were identified when secondary hypertension, including PA, was excluded according to the 2020 International Society of Hypertension Global Hypertension Practice Guidelines (31). Patients underwent thin-slice computer tomography (CT) scan and adrenal venous sampling (AVS) to define unilateral and bilateral PA. AVS was successful when plasma cortisol concentrations were at least three times as high in both adrenal veins as in the inferior vena cava. A lateralization index, defined as the ratio of cortisol-corrected aldosterone from the dominant side to the non-dominant side, of at least 2 was considered to indicate lateralization.

Serum potassium concentration < 3.5 mmol/L was defined as hypokalemia, and urine pH > 7.0 was defined as alkaline urine. Patients were excluded if they have secondary hypertension due to any causes other than PA (such as Cushing syndrome and Grave’s disease); patients with any kind of cancer or history of cancer, diabetes mellitus, thyroid disease, chronic kidney disease (defined as persistently elevated urine albumin excretion ≥30 mg/g [3 mg/mmol] creatinine, persistently reduced estimated glomerular filtration rate (eGFR) < 60 ml/min per 1.73 m^2^, or both, for greater than 3 months) (32), urinary tract infection, and pregnancy were excluded. Patients with missing data were also excluded. The inclusion and exclusion processes are shown in **Supplementary Figures 1**, **2**.

### Data collection

This study selected eligible patients from The First Affiliated Hospital, Sun Yat-sen University, and Sun Yat-sen Memorial Hospital, Sun Yat-sen University. Clinical characteristics including age, gender, systolic blood pressure (SBP), and diastolic blood pressure (DBP) were collected from the hospital information system (HIS). The laboratory tests including serum potassium (K), serum sodium (NA), serum chlorine (CL), serum creatinine (CREA), serum uric acid (UA), serum anion gap (AG), serum calcium (CA), serum total cholesterol (CHOL), serum triglyceride (TG), serum high-density lipoprotein cholesterol (HDL-C), serum low-density lipoprotein cholesterol (LDL-C), and urine pondus hydrogenii (urine pH) were the first test results during the hospitalization and collected from laboratory information system (LIS). All serum specimens were detected by using the automatic biochemical analyzer AU5800 (Beckman Coulter, Brea, CA, USA) with the manufacturer’s reagent kits, while urine specimens were measured by using the automatic urine analyzer COBOI-xs (COBIO, Hungary) with the manufacturer’s reagent kits. Internal quality control was performed daily, and external quality assessment was performed as required.

### Statistical analysis and machine learning-based model development

All statistical analyses were conducted in R software for Windows (Version 3.6.1, https://www.r-project.org/) and the Deepwise and Beckman Coulter DxAI platform (https://dxonline.deepwise.com). Categorical variables were presented as frequencies with percentages, and continuous variables were presented as mean ± standard deviation (SD) or median with interquartile range (IQR). PA group and essential hypertension group from The First Affiliated Hospital, Sun Yat-sen University, were randomly assigned to the training cohort and internal validation cohort, respectively, in a ratio of 7:3. Clinical characteristics and laboratory test results between the PA group and essential hypertension group and between the training cohort and internal validation cohort were compared by using Student’s t-test, Mann–Whitney test, or chi-square test, as appropriate. Feature selection was carried out using R software. Univariate logistic regression analysis was conducted in the training cohort to screen the variables associated with PA. The magnitude of the association was expressed by odds ratio (OR value) with a 95% confidence interval (95% CI). *p* < 0.05 was considered statistically significant. The variables with statistical significance (*p*-value < 0.05) in the previous univariable logistic analysis were selected for a step-backward multivariate logistic regression analysis to identify the independent risk factors (*p* < 0.1) for the prediction of PA. The Hosmer–Lemeshow test was conducted to measure the fitness of the logistic regression model, and *p* > 0.05 means that the model was a good fit. Multicollinearity analysis for all predictor variables was conducted using the R software with the car package, and a variance inflation factor (VIF) of less than 4 means that there is no multicollinearity between predictor variables.

The prediction model was developed using R software with the rms package and displayed online through Deepwise and Beckman Coulter DxAI platform (https://dxonline.deepwise.com/). The model was applied to 1,000 bootstrap resamples in the training cohort for internal validation and external validation to validate the predictive ability. Receiver operating characteristic (ROC) curves were presented to measure the discrimination ability of the prediction model, and areas under the curve (AUCs) were calculated. AUC ranges from 0.5 to 1.0, and a higher value indicates better predictive ability. Calibration curves were conducted to assess how close the model predicted risk is to the actual risk. Decision curve analysis (DCA) was carried out to assess the utility of models for decision making. All reported *p*-values were two-tailed, and *p* < 0.05 was statistically significant.

## Results

### Clinical characteristics of included patients

The study flowchart was presented in **Supplementary Figures 1**, **2**. We firstly obtained 709 patients with PA and 1,215 patients with essential hypertension from The First Affiliated Hospital, Sun Yat-sen University (**Supplementary Figure 1**). According to the above exclusion criteria, a total of 1,314 patients were finally included for analysis, among which 490 patients were PA and 824 patients were essential hypertension. Another 285 patients including 91 patients with PA and 194 patients with essential hypertension were included as the external validation cohort from the Sun Yat-sen Memorial Hospital, Sun Yat-sen University (**Supplementary Figure 2**). The demographic and clinical characteristics of all included patients are summarized in **Table 1**. Patients with PA were older and more frequently female than those with essential hypertension. Significantly increased rates of hypokalemia and alkaline urine were found in patients with PA compared with those with essential hypertension (377/581, 58.0% versus 112/1,018, 11.0% for hypokalemia; 81/581, 13.9% versus 24/1,018, 2.4% for alkaline urine, all *p* < 0.001). Likewise, serum K, serum NA, serum CA, serum UA, AG, serum CHOL, TG, LDL, and serum NA-to-K ratio also showed significant differences between the two groups. However, neither SBP nor DBP showed statistically significant differences.

**Table 1:** Baseline clinical and biochemical characteristics of all patients.

Variable	Essential hypertensionn = 1,018	Primary aldosteronismn = 581	*p*-Value
Age (year) ^#^	45 ± 15	50 ± 12	<0.001^***^
Gender			
Female	389 (38.2%)	299 (51.5%)	<0.001^***^
Male	629 (61.8%)	282 (48.5%)	
SBP (mmHg)^&^	147 (134–160)	148 (135–161)	0.40
DBP (mmHg) ^&^	92 (82–102)	91 (82–100)	0.25
Aldosterone (pg/ml) ^&^	201.78 (148.31–272.31)	307.31 (198.45–505.84)	<0.001^***^
Renin (uIU/ml) ^&^	20.90 (12.50–36.00)	4.20 (2.10–7.30)	<0.001^***^
ARR (pg/ml/uIU/ml) ^&^	9.72 (5.34–17.08)	79.48 (42.06–165.38)	<0.001^***^
K (mmol/L) ^&^	3.92 (3.70–4.13)	3.37 (2.99–3.82)	<0.001^***^
NA (mmol/L) ^&^	140 (139–142)	142 (140–143)	<0.001^***^
CL (mmol/L) ^&^	104 (103–106)	104 (102–106)	0.16
Serum NA-to-K ratio^&^	35.85 (33.82–37.96)	41.94 (36.84–47.8)	<0.001^***^
CREA (mmol/L) ^&^	76 (64–87)	72 (59–88)	0.04^*^
UA (mmol/L) ^&^	399 (334–472)	348 (293–418)	<0.001^***^
AG^&^	14 (13–16)	14 (12–15)	<0.001^***^
CA (mg/dl) ^&^	9.20 (8.84–9.56)	8.96 (8.80–9.20)	<0.001^***^
CHOL (mmol/L) ^&^	4.80 (4.20–5.60)	4.70 (4.00–5.40)	0.005^**^
TG (mmol/L) ^&^	1.44 (1.05–2.03)	1.33 (0.97–1.86)	0.002^**^
HDL-C (mmol/L) ^&^	1.10 (0.94–1.27)	1.09 (0.94–1.29)	0.75
LDL-C (mmol/L) ^&^	3.10 (2.58–3.57)	2.96 (2.43–3.49)	0.006^**^
Alkaline urine (pH > 7) ^&^			
Yes	24 (2.4%)	81 (13.9%)	<0.001^***^
No	994 (97.6%)	500 (86.1%)	
Hypokalemia			
Yes	112 (11.0%)	337 (58.0%)	<0.001^***^
No	906 (89.0%)	244 (42.0%)	

SBP, systolic blood pressure; DBP, diastolic blood pressure; ARR, plasma aldosterone-to-renin-ratio; K, potassium; NA, sodium; CL, chlorine; CREA, creatinine; UA, uric acid; AG, anion gap; CA, calcium; CHOL, cholesterol; TG, triglyceride; HDL-C, high-density lipoprotein cholesterol; LDL-C, low-density lipoprotein cholesterol.

^#^ Data are presented as mean ± SD.

^&^ Data are presented as median (interquartile range).

^*^ p < 0.05; ^**^ p < 0.01; ^***^ p < 0.001.

Then, we randomly divided 1,314 patients into the training cohort and the internal validation cohort in a 70% (919/1,314) to 30% (395/1,314) comparison. The characteristics of the training and internal validation cohorts are summarized in detail in **Supplementary Table 1**. All clinical characteristics showed no statistically significant difference between the training and internal validation cohorts (*p* > 0.05). Clinical and biochemical characteristics of patients in the training cohort, internal validation cohort, and external validation cohort are shown in **Supplementary Table 2**.

### Factors associated with primary aldosteronism in the training cohort

To confirm the possible risk factors of PA, we conducted univariate and multivariate logistic regression analyses in the training cohort. As shown in **Supplementary Table 3**, age (OR: 1.03, 95% CI: 1.02–1.04), gender (OR: 0.46, 95% CI: 0.35–0.60), SBP (OR: 1.008, 95% CI: 1.002–1.014), NA (OR: 1.41, 95% CI: 1.31–1.51), serum NA-to-K ratio (OR: 1.26, 95% CI: 1.22–1.30), K (OR: 0.083, 95% CI: 0.057–0.12), UA (OR: 0.996, 95% CI: 0.994–0.997), AG (OR: 0.91, 95% CI: 0.86–0.96), CHOL (OR: 0.86, 95% CI: 0.75–0.98), TG (OR: 0.84, 95% CI: 0.72–0.96), LDL-C (OR: 0.80, 95% CI: 0.76–0.96), hypokalemia (OR: 10.96, 95% CI: 7.90–15.37), and alkaline urine (urine pH > 7.0) (OR: 6.88, 95% CI: 3.77–13.43) were the candidate risk factors associated with PA occurrence.

A further multivariate logistic analysis shown in **Supplementary Table 4** indicates hypokalemia (OR: 2.09, 95% CI: 1.14–3.82) and alkaline urine (OR: 2.32, 95% CI: 1.01–5.51) were the major risk factors of PA. Some other clinical characteristics, such as age, sex, serum NA, serum NA-to-K ratio, and AG, were also shown as risk factors of PA (age, OR: 1.02, 95% CI: 1.01–1.03; sex, OR: 0.48, 95% CI: 0.34–0.67; serum NA, OR: 1.23, 95% CI: 1.12–1.34; serum NA-to-K ratio, OR: 1.15, 95% CI: 1.09–1.23; and AG, OR: 0.89, 95% CI: 0.84–0.95).

### The prediction model and its performance

**Figure 1:**
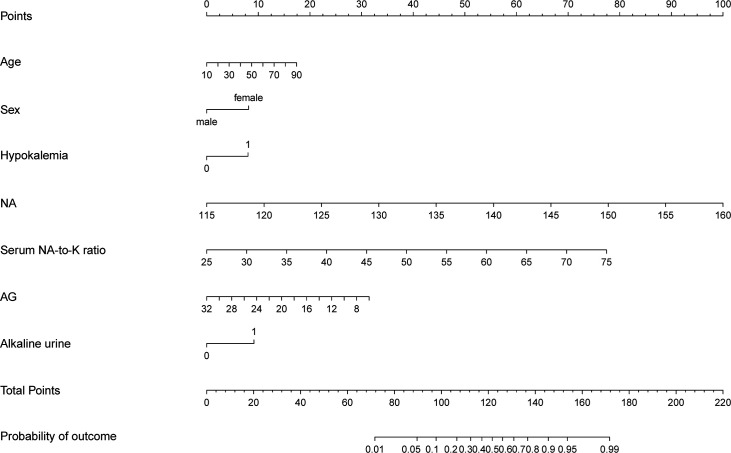
The prediction model presented by nomogram graph. Estimated the probability of primary aldosteronism using the nomogram, located the clinical predictors on each variable axis, and drew the vertical line from that value to the top points scale for calculating the score for each predictor. The total scores from each variable value represent the possibility of primary aldosteronism.

We included the above seven risk factors as the possible predictors of PA to build up a prediction model and presented a nomogram graph as shown in **Figure 1**. Each level of every variable was assigned a score on the points scale. By adding the scores for each of the selected variables, a total score was obtained. Then, the sum score was located on the Total Points scale and vertically projected onto the bottom axis Probability of PA, and thus, a personalized risk of PA can be easily obtained. The threshold value of the model was 54% probability based on the maximal Youden’s index, with a sensitivity of 0.714 and specificity of 0.818. In other words, a risk probability of greater than 54% requires further complicated tests for PA diagnosis. The weight coefficient of each predictor, which represents the contribution of each predictor to the model, is shown in **Supplementary Figure 3**.

**Figure 2:**
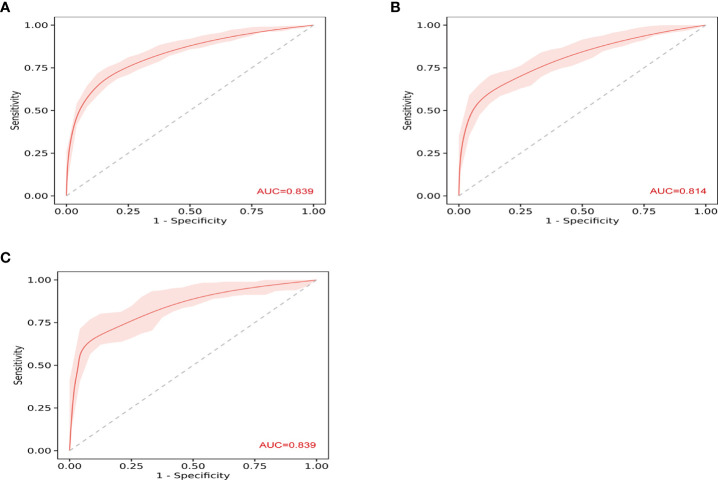
The receiver operating characteristic (ROC) curves show the discriminative ability of prediction model. **(A)** The area under the curve (AUC) in the training set was 0.839 (95% CI: 0.81–0.87). **(B)** The AUC in the internal validation was 0.814 (95% CI: 0.77–0.86). **(C)** The AUC in the external validation was 0.839 (95% CI: 0.79–0.89).

**Figure 3:**
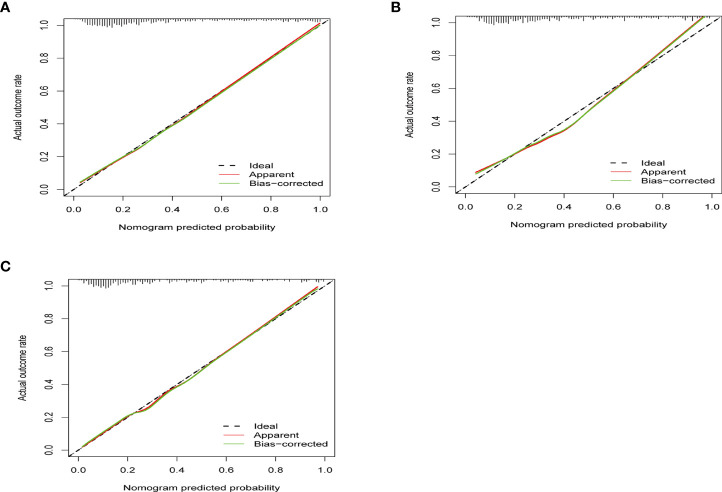
The calibration curves show how close the predicted probability of the model was to the actual, observed probability. **(A)** The calibration curve of training set (*p* = 0.801). **(B)** The calibration curve of internal validation (*p* = 0.302). **(C)** The calibration curve of external validation (*p* = 0.335). x-Axis is the model-predicted probability; y-axis is the actual, observed probability. The black line represents an ideal prediction that the predicted risk was exactly the observed risk. The red line represents the model performance, and the closer the red line was to the ideal line, the better the prediction of the prediction model holds.

The ROC curves in **Figure 2** demonstrated that our model held a very good discriminative ability not only in the training cohort (**Figure 2A**: AUC = 0.839, 95% CI: 0.81–0.87) but also in the internal validation cohort (**Figure 2B**: AUC = 0.814, 95% CI: 0.77–0.86) and the external validation cohort (**Figure 2C**: AUC = 0.839, 95% CI: 0.79–0.89). The *p*-value of the Hosmer–Lemeshow test for this model was 0.122, indicating that the model was well-fitted. As shown in **Figure 3**, the calibration curves visually showed that the model-predicted risk was close to the actual, observed risk. It meant that there were good agreements between the model prediction and the actual observation of PA in hypertensive patients in both the training and validation sets. The prediction performances of our model with a cutoff value of 54% of probability based on the maximal Youden’s index are listed in **Supplementary Table 5** in detail.

DCA was performed to compare the clinical usability and benefits of the prediction model. As shown in **Supplementary Figure 4**, the model showed large benefits in the training cohort (**Supplementary Figure 4A**), the internal validation cohort (**Supplementary Figure 4B**), and the external validation cohort (**Supplementary Figure 4C**). The receiver operating characteristic curves of our model and hypokalemia in predicting PA in all included hypertensive patients (**Supplementary Figures 4D, E**) also indicated that our prediction model was well-fitted.

**Figure 4:**
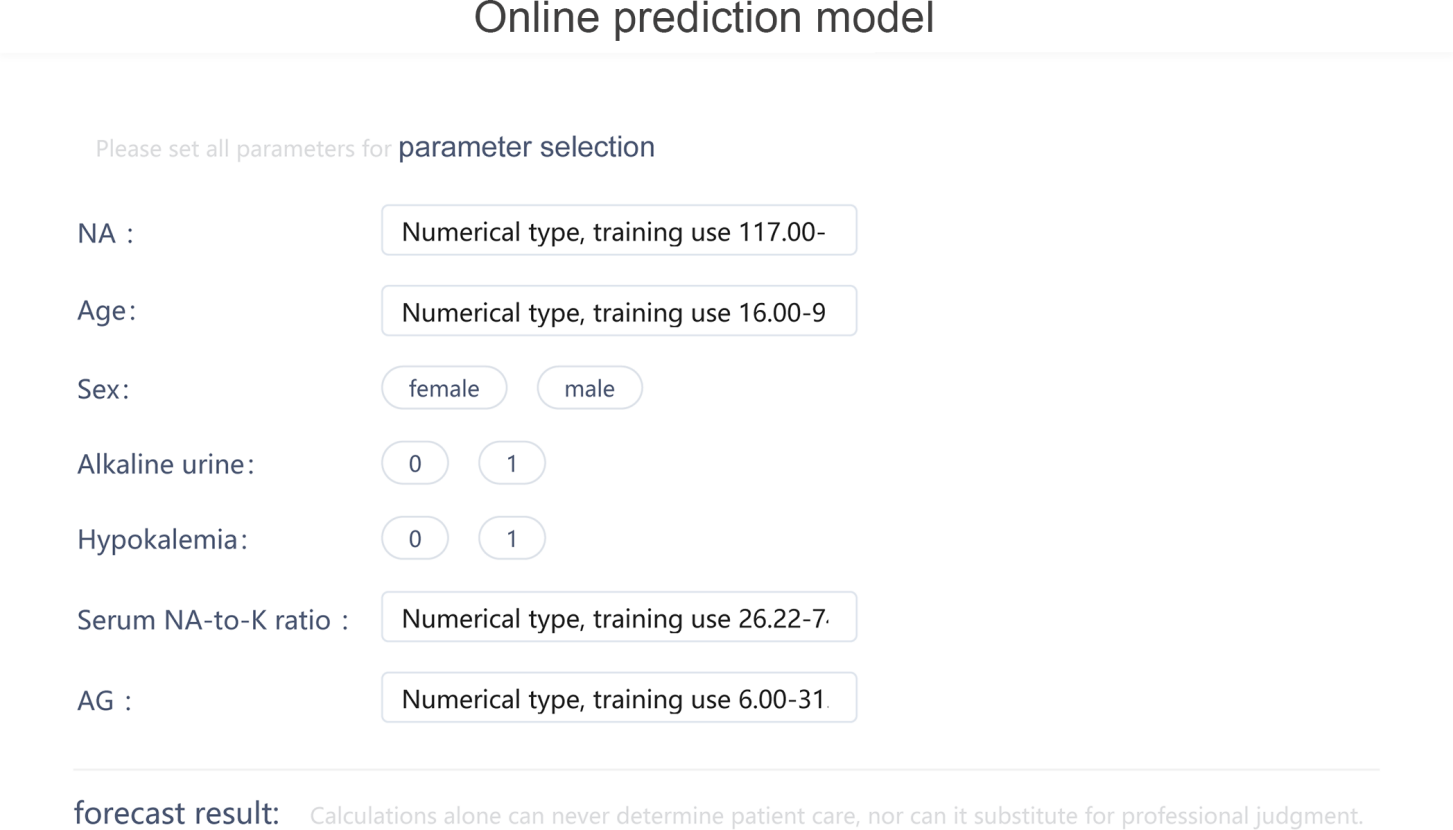
The visualization of the prediction model through Deepwise and Beckman Coulter DxAI platform.

The visualization of the prediction model was displayed online through Deepwise and Beckman Coulter DxAI platform as **Figure 4** (https://dxonline.deepwise.com/prediction/index.html?baseUrl=%2Fapi%2F&id=5244&topicName=undefined&from=share).

### The performance of the model in the diagnosis of unilateral primary aldosteronism

Among all 490 PA patients, only 79 patients had undergone AVS. A total of 29 patients were defined as unilateral PA, and the other 50 patients were diagnosed as bilateral PA. The scores of the unilateral PA tend to be higher than those of the bilateral PA (*p* = 0.002; **Supplementary Figure 5A**). The results showed that our model also performed well for the diagnosis of unilateral PA, with an AUC of 0.786 and high sensitivity of 0.966 (**Supplementary Figure 5B**).

### The performance compared with more selective thresholds (aldosterone > 15 ng/dl or aldosterone-to-renin ratio > 40) and PFK score

Compared to more selective thresholds such as aldosterone > 15 ng/dl or ARR > 40 mentioned in previous studies, the results showed that our model performs as well as using threshold of ARR > 40 (AUC: 0.839 [0.81–0.87] versus 0.868 [0.85–0.89], *p* = 0.091; κ = 231.78, *p* = 0.890) and much better than using aldosterone > 15 ng/dl (AUC: 0.839 [0.81–0.87] versus 0.637 [0.61–0.67], *p* < 0.001; κ = 40.33, *p* < 0.001). When compared with PFK score established by another study, our model exhibited higher AUC (0.839 [0.81–0.87] versus 0.761 [0.73–0.79], *p* < 0.001) than the PFK score (**Supplementary Figure 6**).

## Discussion

In this study, we systematically screened 18 clinical characteristics and laboratory tests that were related to PA and easy to acquire in clinical practice significantly, and finally identified seven risk factors, which are age, sex, serum NA, serum NA-to-K ratio, AG, hypokalemia, and alkaline urine. Further, we successfully developed a novel prediction model based on seven factors to predict the risk of PA and displayed it online for sharing so as to cover more target patients and improve the diagnostic efficiency of PA.

Measuring the ARR for PA screening has been recommended by several guidelines (18, 19, 33), but it is an inconvenience because all guidelines required medications that may interfere with the renin–angiotensin system, or the ARR detection should be withdrawn for some weeks. Moreover, professional equipment for the detection of plasma aldosterone and renin was not available in many medical institutions such as healthcare centers, community hospitals, and even some municipal hospitals in China. Hence, a new prediction model that is portable to use in all medical institutions for predicting PA in patients with hypertension is needed.

The prediction model successfully developed based on the seven factors in our study showed a good discriminative ability in predicting the risk of PA and high clinical usability and benefits for patients. This prediction model is easy and convenient to apply for PA prediction in hypertensive patients, as all factors included are easy to collect in clinical practice. Additionally, the online application of the model improves the sharing level of our model and expands the coverage of target patients. This is conducive to rapid screening of high-risk patients who required further complicated tests for PA diagnosis in the community and saves medical resources. Moreover, the application of the online model can help improve the compliance of PA screening in patients with hypertension, which greatly improves the prognosis of patients with early diagnosis and treatment.

As known, excessive secretion of aldosterone leads to water and sodium retention and excessive excretion of potassium. Thus, in the current study, we tried to combine hypokalemia, serum sodium, anion gap, and serum sodium-to-potassium ratio, which greatly improved the predictive abilities of our models. However, alkaline urine (urine pH > 7.0) has not received attention as a predictor of PA until it was included in a formula to screen the PA in a previous study in Japan (2). The results of our study also indicated that the urine pH test in patients with hypertension might be helpful for prediction ability of the probability of PA. The reason for alkaline urine in patients with PA remains unclear. Some previous studies speculated that alkaline urine may be associated with K depletion (34, 35) or due to transcellular cation exchange of K^+^ and H^+^ (2). Moreover, our model also highlighted age and sex as risk factors. In our study, patients with PA were older and had a higher proportion of women. The reason might be that aging may be associated with an attenuated ability to physiologically secrete aldosterone, as well as an increase in autonomous and pathophysiologic aldosterone secretion (36–38). Women tended to have a high prevalence of somatic mutation for PA occurrence as previously reported (39–41). Nevertheless, both age and sex were found to be vital indicators for PA prediction.

As a prediction tool, our model showed a good performance on the diagnosis of PA in a threshold value of 54%, with AUC values of 0.839, 0.814, and 0.839 and high accuracy of 0.779, 0.749, and 0.793 for training, internal, and external validation sets, respectively. This meant that, in actual clinical practice, our prediction model could accurately diagnose PA patients in hypertensive patients. It would be helpful for clinicians to make a quick recognition of high-risk patients with PA. Additionally, it is undeniable that pinpointing the patients who had unilateral PA would provide further useful information for clinical decisions and patient management as well as resource allocation. Hence, we tried to evaluate the performance of our model for the diagnosis of unilateral PA. The validation results showed that our model also performed well for the diagnosis of unilateral PA, with an AUC of 0.786 and high sensitivity of 96.6% (**Supplementary Figure 5B**). Meanwhile, previous studies had suggested using a minimum plasma aldosterone concentration of 15 ng/dl or an elevated ARR (ARR > 40) for PA screening (42, 43) to achieve a higher specificity and fewer false-positive results. We compared the performance of our model with that of the threshold of ARR > 40 or aldosterone > 15 ng/dl for the diagnosis of PA. The results (**Supplementary Figure 6**) showed that our model performed as well as using a threshold of ARR > 40 (*p* = 0.091) and much better than using a threshold of aldosterone > 15 ng/dl (*p* < 0.001). It appeared that the performance of our model would be the same with more selective thresholds, which met different clinical needs. Moreover, we compared the results with the PFK score established in a previous study (2), which included prediction factors similar to ours. The results shown in **Supplementary Figure 6** suggested that the performance of our model for the diagnosis of PA was much better than that of the PFK score with a higher AUC (AUC: 0.839 [0.810–0.870] versus 0.761 [0.732–0.788], *p* < 0.001).

There were some limitations to our study. Firstly, some indicators like hormones renin or aldosterone estimation were not included in our prediction model, which might affect the accuracy of the model. However, screening PA through ARR is not available in healthcare centers, community hospitals, and even some municipal hospitals in China due to the lack of professional equipment; additionally, the aim of our study was to create a portable model for clinical practice; the indicators that were routine and easily obtained from health examination were considered. Results showed that our prediction model exerted a good discrimination ability of PA recognition. Secondly, although our model exhibited good discrimination ability within cohorts in the study, whether it can be extended to other populations remains unknown. Therefore, further study should be needed to verify our prediction model. Additionally, the prevalence of hypokalemia in our study was about 50%, which was higher than in previous studies. The reason might be most of the patients in our hospital received anti-hypertension treatment for a long time in community healthcare clinics and transferred to our hospital for further treatment because of resistant hypertension and severe hypokalemia. However, this was the most important reason that we conducted this online prediction model, which helped primary care physicians to identify PA and EH early. We included the normal variables in the model, which were all available even in the community healthcare clinics. Our online prediction model showed good discriminative ability and was well-fitted. Finally, as reported in previous studies, the metabolic factors may play an important role in diabetic patients with PA, which was not the same as non-diabetics (44, 45), and similarly, different cancer treatments make the predictors of patients with cancer more complicated (46). Thus, we suppose that it would be better to exclude them when creating the model, which might make the model not applicable for the specific subpopulations.

In conclusion, this study created an online prediction model using a new dataset in the Chinese population to screen PA in patients with hypertension for the first time. This would be helpful for expanding screening for PA in all patients with hypertension and helpful for clinicians for fast screening of hypertensive patients who required further complicated tests for PA diagnosis.

## Perspectives

This is the first study to develop an online prediction model for primary aldosteronism in all patients with hypertension by using common clinical characteristics and laboratory tests. The sharing online of the prediction model allowed it to be widely and portably used by clinicians in hospital and community health services and may help to improve the diagnostic efficiency of primary aldosteronism.

## Data availability statement

The original contributions presented in the study are included in the article/**Supplementary Material**. Further inquiries can be directed to the corresponding authors.

## Ethics statement

The studies involving human participants were reviewed and approved by the Ethics Committee of The First Affiliated Hospital, Sun Yat-sen University and Sun Yat-sen Memorial Hospital, Sun Yat-sen University. Written informed consent was not required for this study, in accordance with the local legislation and institutional requirements.

## Author contributions

WL conceived and designed the study, analyzed the data, and wrote the original draft. WG and PF analyzed the data and revised the manuscript. LZ and ZY prepared the figures and tables. WH conceived and designed the study, funding acquisition, and supervision. PC and NY conducted the formal analysis and revised the manuscript. All authors read and approved the final manuscript.

## Funding

This work was supported by the China Postdoctoral Science Foundation (2021M703708).

## Conflict of interest

The authors declare that the research was conducted in the absence of any commercial or financial relationships that could be construed as a potential conflict of interest.

## Publisher’s note

All claims expressed in this article are solely those of the authors and do not necessarily represent those of their affiliated organizations, or those of the publisher, the editors and the reviewers. Any product that may be evaluated in this article, or claim that may be made by its manufacturer, is not guaranteed or endorsed by the publisher.

## Supplementary material

Supplementary Figure 1 The flow chart of patients screening in training and internal validation cohorts.

Supplementary Figure 2 The flow chars of patients screening in external validation cohort.

Supplementary Figure 3 Weight coefficient of each predictor of the nomogram model.

Supplementary Figure 4 Decision curve analysis of the prediction model in predicting primary aldosteronism (PA). **(A)**. Decision curve analysis in training set. **(B)**. Decision curve analysis in internal validation set. **(C)**. Decision curve analysis in external validation set. The Y-axis measured net benefit, which was calculated by summing the benefits (true positives) and subtracting the harms (false positives). A model was considered of clinical value if it had the highest net benefit compared with other models or simple strategies. **(D)**. and **(E)**. Receiver operating curve analysis of the prediction model and hypokalemia in predicting PA in all hypertension patients (P < 0.001).

Supplementary Figure 5
**(A)**. The score of unilateral PA compared with the bilateral PA. **(B)**. Receiver operating curve analysis of the prediction model in predicting unilateral PA.

Supplementary Figure 6 Receiver operating curve analysis of our model in predicting PA compared with using threshold of ARR>40 or aldosterone>15ng/dL and the PFK score.

Supplementary Table 1 Baseline characteristics of the training set and validation set.

Supplementary Table 2 Baseline parameters and characteristics of all patients in the training set and validation sets.

Supplementary Table 3 Univariate logistic regression analysis of factors potentially associated with primary aldosteronism in the training set.

Supplementary Table 4 Multivariate logistic regression analysis of independent risk factors associated with primary aldosteronism in the training set.

Supplementary Table 5 Prediction performances of models on the training and validation sets.
