# Supplementary material for: Online prediction model for primary aldosteronism in patients with hypertension in Chinese population: A two-center retrospective study
Source: Front Endocrinol (Lausanne). 2022 Aug 2;13:882148. doi: 10.3389/fendo.2022.882148 (PMC9380986; doi:10.3389/fendo.2022.882148)
Supplement: Supplementary Figure 1 — The flow chart of patients screening in training and internal validation cohorts. [file DataSheet_1.zip › Supplementary_Material/Supplementary Table 1.docx]

**Supplementary Table 1. Baseline characteristics of the training set and validation set**

| Variable | Training set  n=919 | Internal Validation set  n=395 | *P* value |
| --- | --- | --- | --- |
| Age (year) ^#^ | 47±14 | 47±14 | 0.76 |
| Gender |  |  |  |
| Female | 384(41.8%) | 179 (45.3%) | 0.24 |
| Male | 535(58.2%) | 216 (54.7%) |  |
| SBP (mmHg) ^&^ | 148(134-161) | 148 (135-160) | 0.86 |
| DBP (mmHg) ^&^ | 93 (82-103) | 92 (82-103) | 0.81 |
| K (mmol/L) ^&^ | 3.78 (3.43-4.05) | 3.82 (3.39-4.07) | 0.41 |
| NA (mmol/L) ^&^ | 141 (139-142) | 141 (139-142) | 0.73 |
| CL (mmol/L) ^&^ | 105 (103-106) | 105 (103-106) | 0.41 |
| Serum NA-to-K ratio^&^ | 37.17 (34.59-41.27) | 36.83 (34.46-41.70) | 0.45 |
| CREA (mmol/L) ^&^ | 74 (59.5-86) | 72 (60-87) | 0.95 |
| UA (mmol/L) ^&^ | 381 (315-448) | 369 (309-447) | 0.24 |
| AG^&^ | 14 (12-16) | 14 (12-16) | 0.39 |
| CA (mg/dL) ^&^ | 9.20 (8.80-9.32) | 9.20 (8.80-9.34) | 0.11 |
| CHOL (mmol/L) ^&^ | 4.80 (4.03-5.50) | 4.80 (4.10-5.60) | 0.28 |
| TG (mmol/L) ^&^ | 1.40 (1.04-1.98) | 1.38 (0.94-1.98) | 0.21 |
| HDL-C (mmol/L) ^&^ | 1.07 (0.93-1.26) | 1.11 (0.94-1.32) | 0.10 |
| LDL-C (mmol/L) ^&^ | 3.02 (2.49-3.50) | 3.06 (2.51-3.54) | 0.41 |
| Alkaline urine (pH >7) |  |  |  |
| Yes | 60 (6.5%) | 27 (6.8%) | 0.84 |
| No | 859(93.5%) | 368 (93.2%) |  |
| Hypokalemia |  |  |  |
| Yes | 272(29.6%) | 119 (30.1%) | 0.85 |
| No | 647(70.4%) | 276 (69.9%) |  |
| Outcome |  |  |  |
| Essential hypertension | 576 (62.7%) | 248 (62.8%) | 0.97 |
| Primary aldosteronism | 343 (37.3%) | 147 (37.2%) |  |

Data are expressed as n (%), Mean ±SD or median (interquartile range). ^#^ denotes that data was presented as Mean ±SD. ^&^ denotes that data was presented as median (interquartile range). SBP, systolic blood pressure; DBP, diastolic blood pressure; K, Potassium; NA, Sodium; CL, Chlorine; CREA, Creatinine; UA, Uric acid; AG, Anion gap; CA, Calcium; CHOL, Cholesterol; TG, Triglyceride; HDL-C, High density lipoprotein cholesterol; LDL-C, Low density lipoprotein cholesterol. ^*^ *P* < 0.05, ^**^ *P* < 0.01, ^***^ *P* < 0.001.
