# Supplementary material for: Online prediction model for primary aldosteronism in patients with hypertension in Chinese population: A two-center retrospective study
Source: Front Endocrinol (Lausanne). 2022 Aug 2;13:882148. doi: 10.3389/fendo.2022.882148 (PMC9380986; doi:10.3389/fendo.2022.882148)
Supplement: Supplementary Figure 1 — The flow chart of patients screening in training and internal validation cohorts. [file DataSheet_1.zip › Supplementary_Material/Supplementary Table 2.docx]

**Supplementary Table 2. Baseline parameters and characteristics of all patients in the training set and validation sets**

|  | Training set | | | Internal validation set | | | External validation set | | |
| --- | --- | --- | --- | --- | --- | --- | --- | --- | --- |
| Variable | Essential  hypertension  n=576 | Primary aldosteronism  n=343 | P value | Essential  hypertension  n=248 | Primary aldosteronism  n=147 | P value | Essential  hypertension  n=194 | Primary aldosteronism  n=91 | P value |
| Age (year) ^#^ | 45±15 | 50±11 | <0.001*** | 45±15 | 50±11 | <0.001*** | 44±15 | 49±12 | 0.004** |
| Gender |  |  |  |  |  |  |  |  |  |
| Female | 200 (34.7%) | 184 (53.8%) | <0.001*** | 106 (42.7%) | 73 (49.7%) | 0.18 | 83 (42.8%) | 42 (46.2%) | 0.59 |
| Male | 376 (65.3%) | 159 (46.4%) |  | 142 (57.3%) | 74 (50.3%) |  | 111 (57.2%) | 49 (53.8%) |  |
| SBP (mmHg)^&^ | 146 (132-159) | 151 (136-164) | 0.002** | 150 (135-161) | 145 (134-158) | 0.12 | 146 (136-162) | 142 (131-152) | 0.028* |
| DBP (mmHg) ^&^ | 93 (82-103) | 93 (83-102) | 0.84 | 93 (84-103) | 92 (81-100) | 0.089 | 89 (80-98) | 85 (78-96) | 0.14 |
| K (mmol/L) ^&^ | 3.90 (3.68-4.11) | 3.35 (2.94-3.79) | <0.001*** | 3.92 (3.72-4.13) | 3.35 (3.05-3.84) | <0.001*** | 3.96 (3.76-4.18) | 3.47 (3.17-3.83) | <0.001*** |
| NA (mmol/L) ^&^ | 140 (139-142) | 142 (140-143) | <0.001*** | 140 (139-142) | 142 (141-144) | <0.001*** | 140 (138-142) | 141 (140-143) | <0.001*** |
| CL (mmol/L) ^&^ | 105 (103-106) | 105 (102-106) | 0.95 | 104 (102-106) | 105 (103-106.5) | 0.21 | 103 (102-105) | 104 (101.5-105) | 0.20 |
| Serum NA-to-K ratio^&^ | 36.03 (34.07-38.22) | 42.37 (37.05-48.73) | <0.001*** | 35.87 (33.82-37.97) | 42.11 (36.83-46.84) | <0.001*** | 35.15 (33.17-37.39) | 40.46 (36.54-44.36) | <0.001*** |
| CREA (mmol/L) ^&^ | 76 (63-87) | 70 (56-86) | 0.004** | 72 (60-85) | 73 (59-88) | 0.64 | 80 (71-92) | 83 (69-96) | 0.64 |
| UA (mmol/L) ^&^ | 399 (331-472) | 349 (295-416) | <0.001*** | 388 (334-458) | 337 (291-414) | <0.001*** | 415 (338.5-488) | 363 (297-438.25) | 0.003** |
| AG^&^ | 14 (13-16) | 14 (12-15) | 0.001** | 14 (13-16) | 14 (12-15) | 0.055 | 16 (14-18) | 14 (13-16) | <0.001*** |
| CA (mg/dL) ^&^ | 9.20 (8.80-9.49) | 8.96 (8.80-9.20) | <0.001*** | 9.20 (8.80-9.60) | 8.88 (8.66-9.20) | <0.001*** | 9.36 (9.16-9.60) | 9.20 (8.92-9.48) | <0.001*** |
| CHOL (mmol/L) ^&^ | 4.80 (4.10-5.50) | 4.60 (3.90-5.40) | 0.019* | 4.90 (4.20-5.60) | 4.70 (3.90-5.45) | 0.092 | 4.89 (4.27-5.59) | 4.91 (4.19-5.56) | 0.74 |
| TG (mmol/L) ^&^ | 1.44 (1.07-2.02) | 1.35 (0.98-1.83) | 0.044* | 1.43 (0.96-2.03) | 1.22 (0.92-1.86) | 0.044* | 1.46 (1.05-2.11) | 1.30 (1.03-1.98) | 0.26 |
| HDL-C (mmol/L) ^&^ | 1.07 (0.93-1.24) | 1.07 (0.93-1.29) | 0.56 | 1.11 (0.94-1.31) | 1.11 (0.93-1.35) | 0.43 | 1.13 (1.00-1.33) | 1.10 (1.00-1.27) | 0.30 |
| LDL-C (mmol/L) ^&^ | 3.08 (2.55-3.54) | 2.89 (2.41-3.45) | 0.018* | 3.11 (2.62-3.54) | 2.90 (2.43-3.51) | 0.066 | 3.13 (2.62-3.64) | 3.17 (2.56-3.63) | 0.94 |
| Alkaline urine (pH >7) ^&^ |  |  |  |  |  |  |  |  |  |
| Yes | 13 (2.3%) | 47 (13.7%) | <0.001*** | 5 (2.0%) | 22 (15.0%) | <0.001*** | 6 (3.1%) | 12 (13.2%) | 0.001** |
| No | 563 (97.7%) | 296 (86.3%) |  | 243 (98.0%) | 125 (85.0%) |  | 188 (96.9%) | 79 (86.8%) |  |
| Hypokalemia |  |  |  |  |  |  |  |  |  |
| Yes | 68 (11.8%) | 204 (59.5%) | <0.001*** | 34 (13.7%) | 85 (57.8%) | <0.001*** | 10 (5.2%) | 48 (52.7%) | <0.001*** |
| No | 508 (88.2%) | 139 (40.5%) |  | 214 (86.3%) | 62 (42.2%) |  | 184 (94.8%) | 43 (47.3%) |  |

Data are expressed as n (%), Mean ±SD or median (interquartile range). ^#^ denotes that data was presented as Mean ±SD. ^&^ denotes that data was presented as median (interquartile range). SBP, systolic blood pressure; DBP, diastolic blood pressure; K, Potassium; NA, Sodium; CL, Chlorine; CREA, Creatinine; UA, Uric acid; AG, Anion gap; CA, Calcium; CHOL, Cholesterol; TG, Triglyceride; HDL-C, High density lipoprotein cholesterol; LDL-C, Low density lipoprotein cholesterol. ^*^ *P* < 0.05, ^**^ *P* < 0.01, ^***^ *P* < 0.001.
