# Supplementary material for: Online prediction model for primary aldosteronism in patients with hypertension in Chinese population: A two-center retrospective study
Source: Front Endocrinol (Lausanne). 2022 Aug 2;13:882148. doi: 10.3389/fendo.2022.882148 (PMC9380986; doi:10.3389/fendo.2022.882148)
Supplement: Supplementary Figure 1 — The flow chart of patients screening in training and internal validation cohorts. [file DataSheet_1.zip › Supplementary_Material/Supplementary Table 3.docx]

**Supplementary Table 3. Univariate logistic regression analysis of factors potentially associated with primary aldosteronism in the training set**

| Variable | OR (95% CI) | *P* value |
| --- | --- | --- |
| Age (year) | 1.03 (1.02-1.04) | <0.001^***^ |
| Gender | 0.46 (0.35-0.60) | <0.001^***^ |
| Female |  |  |
| Male |  |  |
| SBP (mmHg) | 1.008 (1.002-1.014) | 0.018^*^ |
| DBP (mmHg) | 1.00 (0.90-1.01) | 0.90 |
| K (mmol/L) | 0.083 (0.057-0.12) | <0.001^***^ |
| NA (mmol/L) | 1.41 (1.31-1.51) | <0.001^***^ |
| CL (mmol/L) | 0.99 (0.97-1.02) | 0.67 |
| Serum NA-to-K ratio | 1.26 (1.22-1.30) | <0.001^***^ |
| CREA (mmol/L) | 1.00 (0.998-1.005) | 0.37 |
| UA (mmol/L) | 0.996 (0.994-0.997) | <0.001^***^ |
| AG | 0.91 (0.86-0.96) | <0.001^***^ |
| CA (mg/dL) | 1.01 (0.99-1.21) | 0.09 |
| CHOL (mmol/L) | 0.86 (0.75-0.98) | 0.022^*^ |
| TG (mmol/L) | 0.84(0.72-0.96) | 0.016^*^ |
| HDL-C (mmol/L) | 1.29 (0.76-2.17) | 0.35 |
| LDL-C (mmol/L) | 0.80 (0.67-0.96) | 0.018^*^ |
| Alkaline urine (pH >7) | 6.88 (3.77-13.43) | <0.001^***^ |
| Yes |  |  |
| No |  |  |
| Hypokalemia | 10.96 (7.90-15.37) | <0.001^***^ |
| Yes |  |  |
| No |  |  |

SBP, systolic blood pressure; DBP, diastolic blood pressure; K, Potassium; NA, Sodium; CL, Chlorine; CREA, Creatinine; UA, Uric acid; AG, Anion gap; CA, Calcium; CHOL, Cholesterol; TG, Triglyceride; HDL-C, High density lipoprotein cholesterol; LDL-C, Low density lipoprotein cholesterol; CI, confidence interval. ^*^ *P* < 0.05, ^**^ *P* < 0.01, ^***^ *P* < 0.001.
