# Supplementary material for: Online prediction model for primary aldosteronism in patients with hypertension in Chinese population: A two-center retrospective study
Source: Front Endocrinol (Lausanne). 2022 Aug 2;13:882148. doi: 10.3389/fendo.2022.882148 (PMC9380986; doi:10.3389/fendo.2022.882148)
Supplement: Supplementary Figure 1 — The flow chart of patients screening in training and internal validation cohorts. [file DataSheet_1.zip › Supplementary_Material/Supplementary Table 4.docx]

**Supplementary Table** **4. Multivariate logistic regression analysis of independent risk factors associated with primary aldosteronism in the training set**

| Variable | Regression coefficient | Adjusted OR (95% CI) | *P* value |
| --- | --- | --- | --- |
| Intercept | -34.11 |  | <0.001^***^ |
| Age | 0.02 | 1.02 (1.01-1.03) | 0.002^**^ |
| Sex (male) | -0.74 | 0.48 (0.34-0.67) | <0.001^***^ |
| Hypokalemia | 0.74 | 2.09 (1.14-3.82) | 0.02* |
| NA | 0.20 | 1.23 (1.12-1.34) | <0.001^***^ |
| Serum NA-to-K ratio | 0.14 | 1.15 (1.09-1.23) | <0.001^***^ |
| AG | -0.11 | 0.89 (0.84-0.95) | <0.001^***^ |
| Alkaline urine (pH>7.0) | 0.84 | 2.32 (1.01-5.51) | 0.051 |

Only variables with *P* < 0.1 in the univariate analysis are shown in the table. NA, Sodium; UA, Uric acid; AG, Anion gap; CI, confidence interval. ^*^ *P* < 0.05, ^**^ *P* < 0.01, ^***^ *P* < 0.001.
