# Supplementary material for: Online prediction model for primary aldosteronism in patients with hypertension in Chinese population: A two-center retrospective study
Source: Front Endocrinol (Lausanne). 2022 Aug 2;13:882148. doi: 10.3389/fendo.2022.882148 (PMC9380986; doi:10.3389/fendo.2022.882148)
Supplement: Supplementary Figure 1 — The flow chart of patients screening in training and internal validation cohorts. [file DataSheet_1.zip › Supplementary_Material/Supplementary Table 5.docx]

**Supplementary Table 5. Prediction performances of models on the training and validation sets**

|  | AUC (95%CI) | Sensitivity | Specificity | Accuracy | PPV | NPV |
| --- | --- | --- | --- | --- | --- | --- |
| Training set | 0.839 (0.81-0.87) | 0.714 | 0.818 | 0.779 | 0.700 | 0.828 |
| Internal validation set | 0.814 (0.77-0.86) | 0.680 | 0.790 | 0.749 | 0.658 | 0.807 |
| External validation set | 0.839 (0.79-0.89) | 0.582 | 0.892 | 0.793 | 0.716 | 0.820 |

AUC, Area under curve; CI, Confidence interval; PPV, Positive predictive value; NPV, Negative predictive value.
