# Supplementary material for: Online prediction model for primary aldosteronism in patients with hypertension in Chinese population: A two-center retrospective study
Source: Front Endocrinol (Lausanne). 2022 Aug 2;13:882148. doi: 10.3389/fendo.2022.882148 (PMC9380986; doi:10.3389/fendo.2022.882148)
Supplement: Supplementary Figure 1 — The flow chart of patients screening in training and internal validation cohorts. [file DataSheet_1.zip › Supplementary_Material/Supplementary_Material.docx]

Supplementary Material

# Supplementary Method

Detail of logistic regression model:

1. The data set was randomly divided into training set and validation set at a ratio of 7:3. and the Pearson correlation analysis with *r* ≥0.90 was used to eliminate the redundancy.
2. Univariate logistic regression analysis was conducted in the training cohort to screen the variables associated with PA. The magnitude of the association was expressed by odds ratio (OR value) with 95% confidence interval (95% CI). *P* < 0.05 was considered as statistically significant.
3. The variables with statistical significance (*P* < 0.05) in the previous univariable logistic analysis were selected into a step-backward multivariate logistic regression analysis to identify the independent risk factors (*P* < 0.1) for prediction of PA.
4. Logistic regression algorithm was used to train the model.

In logistic regression, input features are linearly scaled just as with linear regression; however, the result is then fed as an input to the logistic function (as below). This function provides a nonlinear transformation on its input and ensures that the range of the output, which is interpreted as the probability of the input belonging to class 1, lies in the interval [0,1]. For logistic regression, we found our coefficients by maximizing the likelihood of the data.


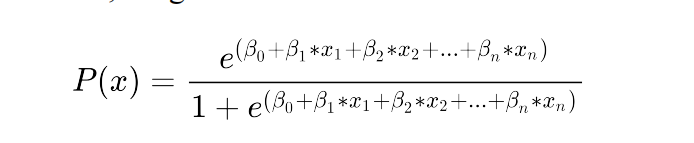


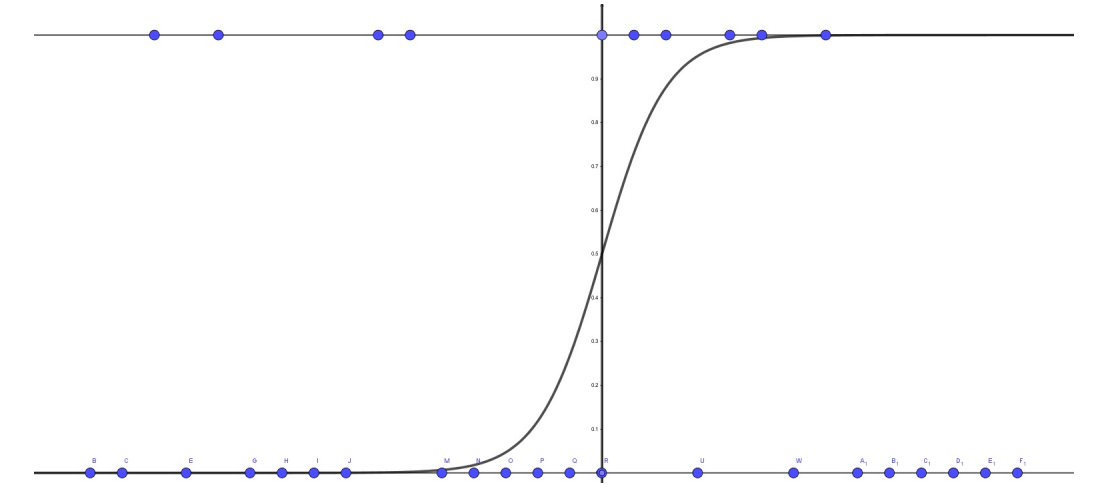


Besides, we might apply regularization to avoid overfitting. In Ridge regression(L2-norm), the regression coefficients are shrunk by introducing a penalty, as follows:


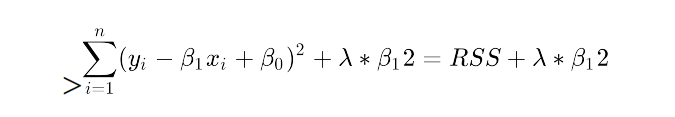


1. Model evaluation

We used the area under the curve (AUC), accuracy, specificity, and sensitivity to evaluate the predictive performance of our model. We also used calibration curves and decision curve analysis to evaluate the discriminative ability and clinical suability of the model. The performance of the model was tested on the external validation set.

# Supplementary Figures and Tables

## Supplementary Figures





**Supplementary Figure 1.** The flow chart of patients screening in training and internal validation cohorts.





**Supplementary Figure 2.** The flow chars of patients screening in external validation cohort.


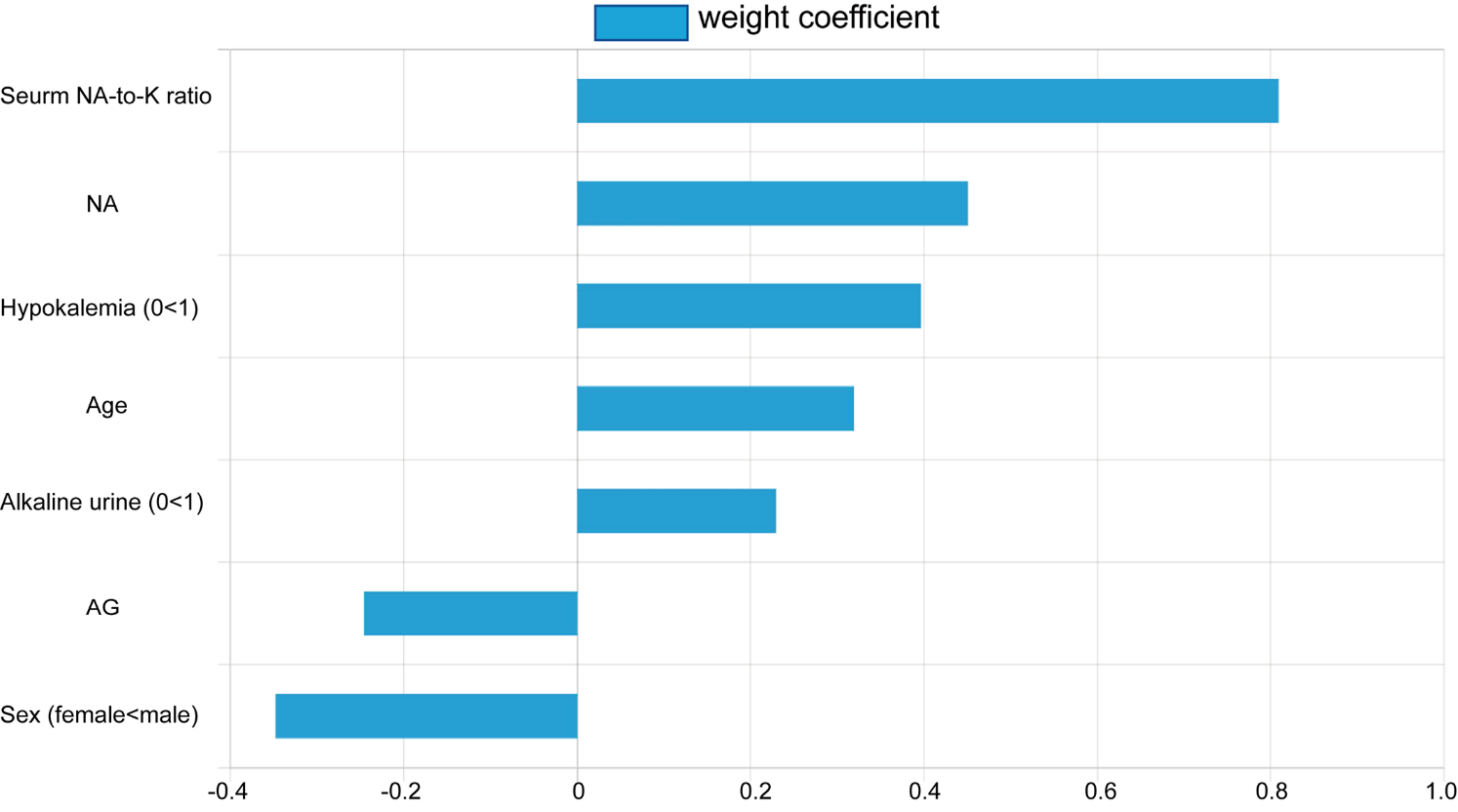


**Supplementary Figure 3.** Weight coefficient of each predictor of the nomogram model.


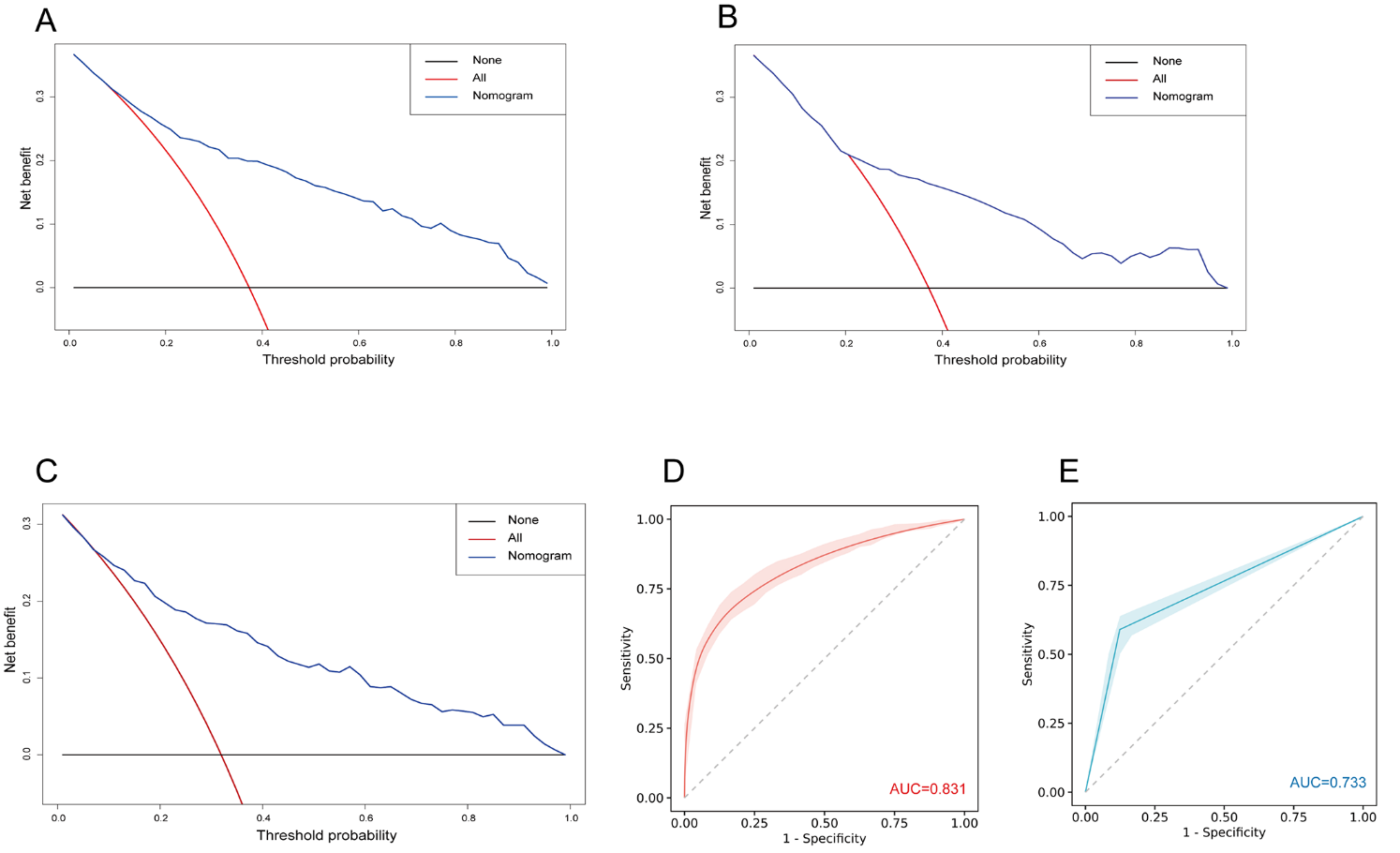


**Supplementary Figure 4. Decision curve analysis of the prediction model in predicting primary aldosteronism (PA).** **A.** Decision curve analysis in training set. **B.** Decision curve analysis in internal validation set. **C.** Decision curve analysis o in external validation set. The Y-axis measured net benefit, which was calculated by summing the benefits (true positives) and subtracting the harms (false positives). A model was considered of clinical value if it had the highest net benefit compared with other models or simple strategies. **D.** and **E.** Receiver operating curve analysis of the prediction model and hypokalemia in predicting PA in all hypertension patients (*P* < 0.001).


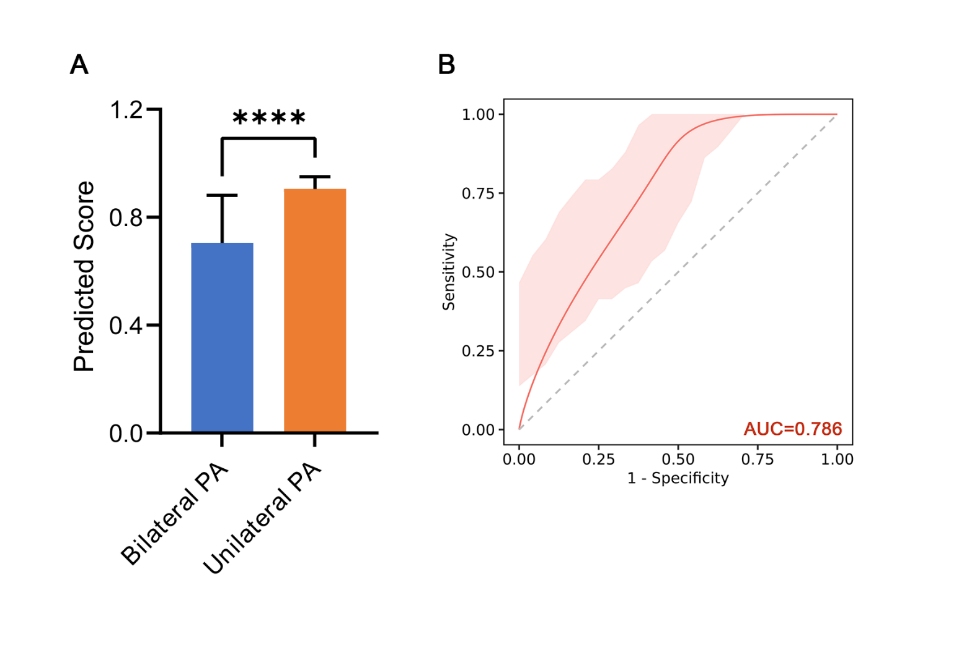


**Supplementary Figure 5. A.** The score of unilateral PA compared with the bilateral PA. **B.** Receiver operating curve analysis of the prediction model in predicting unilateral PA.

**
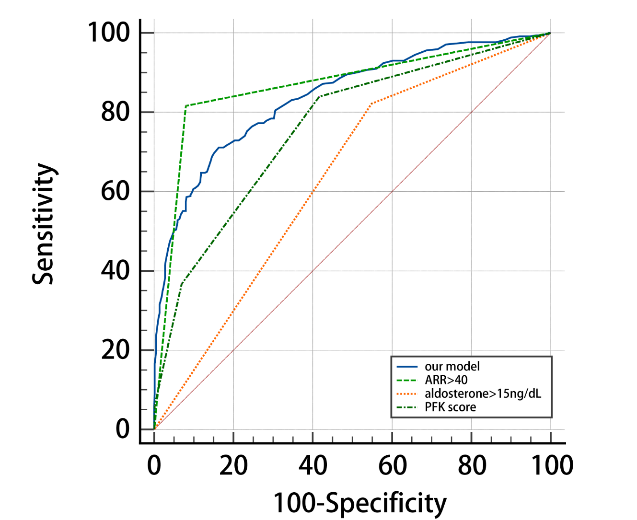
**

**Supplementary Figure 6.** Receiver operating curve analysis of our model in predicting PA compared with using threshold of ARR > 40 or aldosterone > 15ng/dL and the PFK score.

## Supplementary Tables

**Supplementary Table 1.** Baseline characteristics of the training set and validation set

| Variable | Training set  n=919 | Internal Validation set  n=395 | *P* value |
| --- | --- | --- | --- |
| Age (year) ^#^ | 47±14 | 47±14 | 0.76 |
| Gender |  |  |  |
| Female | 384(41.8%) | 179 (45.3%) | 0.24 |
| Male | 535(58.2%) | 216 (54.7%) |  |
| SBP (mmHg) ^&^ | 148(134-161) | 148 (135-160) | 0.86 |
| DBP (mmHg) ^&^ | 93 (82-103) | 92 (82-103) | 0.81 |
| K (mmol/L) ^&^ | 3.78 (3.43-4.05) | 3.82 (3.39-4.07) | 0.41 |
| NA (mmol/L) ^&^ | 141 (139-142) | 141 (139-142) | 0.73 |
| CL (mmol/L) ^&^ | 105 (103-106) | 105 (103-106) | 0.41 |
| Serum NA-to-K ratio^&^ | 37.17 (34.59-41.27) | 36.83 (34.46-41.70) | 0.45 |
| CREA (mmol/L) ^&^ | 74 (59.5-86) | 72 (60-87) | 0.95 |
| UA (mmol/L) ^&^ | 381 (315-448) | 369 (309-447) | 0.24 |
| AG^&^ | 14 (12-16) | 14 (12-16) | 0.39 |
| CA (mg/dL) ^&^ | 9.20 (8.80-9.32) | 9.20 (8.80-9.34) | 0.11 |
| CHOL (mmol/L) ^&^ | 4.80 (4.03-5.50) | 4.80 (4.10-5.60) | 0.28 |
| TG (mmol/L) ^&^ | 1.40 (1.04-1.98) | 1.38 (0.94-1.98) | 0.21 |
| HDL-C (mmol/L) ^&^ | 1.07 (0.93-1.26) | 1.11 (0.94-1.32) | 0.10 |
| LDL-C (mmol/L) ^&^ | 3.02 (2.49-3.50) | 3.06 (2.51-3.54) | 0.41 |
| Alkaline urine (pH >7) |  |  |  |
| Yes | 60 (6.5%) | 27 (6.8%) | 0.84 |
| No | 859(93.5%) | 368 (93.2%) |  |
| Hypokalemia |  |  |  |
| Yes | 272(29.6%) | 119 (30.1%) | 0.85 |
| No | 647(70.4%) | 276 (69.9%) |  |
| Outcome |  |  |  |
| Essential hypertension | 576 (62.7%) | 248 (62.8%) | 0.97 |
| Primary aldosteronism | 343 (37.3%) | 147 (37.2%) |  |

Data are expressed as n (%), Mean ±SD or median (interquartile range). ^#^ denotes that data was presented as Mean ±SD. ^&^ denotes that data was presented as median (interquartile range). SBP, systolic blood pressure; DBP, diastolic blood pressure; K, Potassium; NA, Sodium; CL, Chlorine; CREA, Creatinine; UA, Uric acid; AG, Anion gap; CA, Calcium; CHOL, Cholesterol; TG, Triglyceride; HDL-C, High density lipoprotein cholesterol; LDL-C, Low density lipoprotein cholesterol. ^*^ *P* < 0.05, ^**^ *P* < 0.01, ^***^ *P* < 0.001.

**Supplementary Table 2.** Baseline parameters and characteristics of all patients in the training set and validation sets

|  | Training set | | | Internal validation set | | | External validation set | | |
| --- | --- | --- | --- | --- | --- | --- | --- | --- | --- |
| Variable | Essential  hypertension  n=576 | Primary aldosteronism  n=343 | P value | Essential  hypertension  n=248 | Primary aldosteronism  n=147 | P value | Essential  hypertension  n=194 | Primary aldosteronism  n=91 | P value |
| Age (year) ^#^ | 45±15 | 50±11 | <0.001*** | 45±15 | 50±11 | <0.001*** | 44±15 | 49±12 | 0.004** |
| Gender |  |  |  |  |  |  |  |  |  |
| Female | 200 (34.7%) | 184 (53.8%) | <0.001*** | 106 (42.7%) | 73 (49.7%) | 0.18 | 83 (42.8%) | 42 (46.2%) | 0.59 |
| Male | 376 (65.3%) | 159 (46.4%) |  | 142 (57.3%) | 74 (50.3%) |  | 111 (57.2%) | 49 (53.8%) |  |
| SBP (mmHg)^&^ | 146 (132-159) | 151 (136-164) | 0.002** | 150 (135-161) | 145 (134-158) | 0.12 | 146 (136-162) | 142 (131-152) | 0.028* |
| DBP (mmHg) ^&^ | 93 (82-103) | 93 (83-102) | 0.84 | 93 (84-103) | 92 (81-100) | 0.089 | 89 (80-98) | 85 (78-96) | 0.14 |
| K (mmol/L) ^&^ | 3.90 (3.68-4.11) | 3.35 (2.94-3.79) | <0.001*** | 3.92 (3.72-4.13) | 3.35 (3.05-3.84) | <0.001*** | 3.96 (3.76-4.18) | 3.47 (3.17-3.83) | <0.001*** |
| NA (mmol/L) ^&^ | 140 (139-142) | 142 (140-143) | <0.001*** | 140 (139-142) | 142 (141-144) | <0.001*** | 140 (138-142) | 141 (140-143) | <0.001*** |
| CL (mmol/L) ^&^ | 105 (103-106) | 105 (102-106) | 0.95 | 104 (102-106) | 105 (103-106.5) | 0.21 | 103 (102-105) | 104 (101.5-105) | 0.20 |
| Serum NA-to-K ratio^&^ | 36.03 (34.07-38.22) | 42.37 (37.05-48.73) | <0.001*** | 35.87 (33.82-37.97) | 42.11 (36.83-46.84) | <0.001*** | 35.15 (33.17-37.39) | 40.46 (36.54-44.36) | <0.001*** |
| CREA (mmol/L) ^&^ | 76 (63-87) | 70 (56-86) | 0.004** | 72 (60-85) | 73 (59-88) | 0.64 | 80 (71-92) | 83 (69-96) | 0.64 |
| UA (mmol/L) ^&^ | 399 (331-472) | 349 (295-416) | <0.001*** | 388 (334-458) | 337 (291-414) | <0.001*** | 415 (338.5-488) | 363 (297-438.25) | 0.003** |
| AG^&^ | 14 (13-16) | 14 (12-15) | 0.001** | 14 (13-16) | 14 (12-15) | 0.055 | 16 (14-18) | 14 (13-16) | <0.001*** |
| CA (mg/dL) ^&^ | 9.20 (8.80-9.49) | 8.96 (8.80-9.20) | <0.001*** | 9.20 (8.80-9.60) | 8.88 (8.66-9.20) | <0.001*** | 9.36 (9.16-9.60) | 9.20 (8.92-9.48) | <0.001*** |
| CHOL (mmol/L) ^&^ | 4.80 (4.10-5.50) | 4.60 (3.90-5.40) | 0.019* | 4.90 (4.20-5.60) | 4.70 (3.90-5.45) | 0.092 | 4.89 (4.27-5.59) | 4.91 (4.19-5.56) | 0.74 |
| TG (mmol/L) ^&^ | 1.44 (1.07-2.02) | 1.35 (0.98-1.83) | 0.044* | 1.43 (0.96-2.03) | 1.22 (0.92-1.86) | 0.044* | 1.46 (1.05-2.11) | 1.30 (1.03-1.98) | 0.26 |
| HDL-C (mmol/L) ^&^ | 1.07 (0.93-1.24) | 1.07 (0.93-1.29) | 0.56 | 1.11 (0.94-1.31) | 1.11 (0.93-1.35) | 0.43 | 1.13 (1.00-1.33) | 1.10 (1.00-1.27) | 0.30 |
| LDL-C (mmol/L) ^&^ | 3.08 (2.55-3.54) | 2.89 (2.41-3.45) | 0.018* | 3.11 (2.62-3.54) | 2.90 (2.43-3.51) | 0.066 | 3.13 (2.62-3.64) | 3.17 (2.56-3.63) | 0.94 |
| Alkaline urine (pH >7) ^&^ |  |  |  |  |  |  |  |  |  |
| Yes | 13 (2.3%) | 47 (13.7%) | <0.001*** | 5 (2.0%) | 22 (15.0%) | <0.001*** | 6 (3.1%) | 12 (13.2%) | 0.001** |
| No | 563 (97.7%) | 296 (86.3%) |  | 243 (98.0%) | 125 (85.0%) |  | 188 (96.9%) | 79 (86.8%) |  |
| Hypokalemia |  |  |  |  |  |  |  |  |  |
| Yes | 68 (11.8%) | 204 (59.5%) | <0.001*** | 34 (13.7%) | 85 (57.8%) | <0.001*** | 10 (5.2%) | 48 (52.7%) | <0.001*** |
| No | 508 (88.2%) | 139 (40.5%) |  | 214 (86.3%) | 62 (42.2%) |  | 184 (94.8%) | 43 (47.3%) |  |

Data are expressed as n (%), Mean ± SD or median (interquartile range). ^#^ denotes that data was presented as Mean ±SD. ^&^ denotes that data was presented as median (interquartile range). SBP, systolic blood pressure; DBP, diastolic blood pressure; K, Potassium; NA, Sodium; CL, Chlorine; CREA, Creatinine; UA, Uric acid; AG, Anion gap; CA, Calcium; CHOL, Cholesterol; TG, Triglyceride; HDL-C, High density lipoprotein cholesterol; LDL-C, Low density lipoprotein cholesterol. ^*^ *P* < 0.05, ^**^ *P* < 0.01, ^***^ *P* < 0.001.

**Supplementary Table 3.** Univariate logistic regression analysis of factors potentially associated with primary aldosteronism in the training set

| Variable | OR (95% CI) | *P* value |
| --- | --- | --- |
| Age (year) | 1.03 (1.02-1.04) | <0.001^***^ |
| Gender | 0.46 (0.35-0.60) | <0.001^***^ |
| Female |  |  |
| Male |  |  |
| SBP (mmHg) | 1.008 (1.002-1.014) | 0.018^*^ |
| DBP (mmHg) | 1.00 (0.90-1.01) | 0.90 |
| K (mmol/L) | 0.083 (0.057-0.12) | <0.001^***^ |
| NA (mmol/L) | 1.41 (1.31-1.51) | <0.001^***^ |
| CL (mmol/L) | 0.99 (0.97-1.02) | 0.67 |
| Serum NA-to-K ratio | 1.26 (1.22-1.30) | <0.001^***^ |
| CREA (mmol/L) | 1.00 (0.998-1.005) | 0.37 |
| UA (mmol/L) | 0.996 (0.994-0.997) | <0.001^***^ |
| AG | 0.91 (0.86-0.96) | <0.001^***^ |
| CA (mg/dL) | 1.01 (0.99-1.21) | 0.09 |
| CHOL (mmol/L) | 0.86 (0.75-0.98) | 0.022^*^ |
| TG (mmol/L) | 0.84(0.72-0.96) | 0.016^*^ |
| HDL-C (mmol/L) | 1.29 (0.76-2.17) | 0.35 |
| LDL-C (mmol/L) | 0.80 (0.67-0.96) | 0.018^*^ |
| Alkaline urine (pH >7) | 6.88 (3.77-13.43) | <0.001^***^ |
| Yes |  |  |
| No |  |  |
| Hypokalemia | 10.96 (7.90-15.37) | <0.001^***^ |
| Yes |  |  |
| No |  |  |

SBP, systolic blood pressure; DBP, diastolic blood pressure; K, Potassium; NA, Sodium; CL, Chlorine; CREA, Creatinine; UA, Uric acid; AG, Anion gap; CA, Calcium; CHOL, Cholesterol; TG, Triglyceride; HDL-C, High density lipoprotein cholesterol; LDL-C, Low density lipoprotein cholesterol; CI, confidence interval. ^*^ *P* < 0.05, ^**^ *P* < 0.01, ^***^ *P* < 0.001.

**Supplementary Table 4.** Multivariate logistic regression analysis of independent risk factors associated with primary aldosteronism in the training set

| Variable | Regression coefficient | Adjusted OR (95% CI) | *P* value |
| --- | --- | --- | --- |
| Intercept | -34.11 |  | <0.001^***^ |
| Age | 0.02 | 1.02 (1.01-1.03) | 0.002^**^ |
| Sex (male) | -0.74 | 0.48 (0.34-0.67) | <0.001^***^ |
| Hypokalemia | 0.74 | 2.09 (1.14-3.82) | 0.02* |
| NA | 0.20 | 1.23 (1.12-1.34) | <0.001^***^ |
| Serum NA-to-K ratio | 0.14 | 1.15 (1.09-1.23) | <0.001^***^ |
| AG | -0.11 | 0.89 (0.84-0.95) | <0.001^***^ |
| Alkaline urine (pH>7.0) | 0.84 | 2.32 (1.01-5.51) | 0.051 |

Only variables with *P*<0.1 in the univariate analysis are shown in the table. NA, Sodium; UA, Uric acid; AG, Anion gap; CI, confidence interval. ^*^ *P* < 0.05, ^**^ *P* < 0.01, ^***^ *P* < 0.001.

**Supplementary Table 5.** Prediction performances of models on the training and validation sets

|  | AUC (95%CI) | Sensitivity | Specificity | Accuracy | PPV | NPV |
| --- | --- | --- | --- | --- | --- | --- |
| Training set | 0.839 (0.81-0.87) | 0.714 | 0.818 | 0.779 | 0.700 | 0.828 |
| Internal validation set | 0.814 (0.77-0.86) | 0.680 | 0.790 | 0.749 | 0.658 | 0.807 |
| External validation set | 0.839 (0.79-0.89) | 0.582 | 0.892 | 0.793 | 0.716 | 0.820 |

AUC, Area under curve; CI, Confidence interval; PPV, Positive predictive value; NPV, Negative predictive value.
